# Studying the Effects of Collagenase (Type 1) on the Collagen in Woody Breast Meat

**DOI:** 10.3390/ani10091602

**Published:** 2020-09-09

**Authors:** Amit Morey, Meredith Lane Johnson, Jasmine Kataria, John Michael Gonzalez

**Affiliations:** 1 Department of Poultry Science, 201 Poultry Science Bldg, 260 Lem Morrison Dr., Auburn University, Auburn, AL 36849, USA; mlj0022@tigermail.auburn.edu (M.L.J.); jasmine.k@uga.edu (J.K.); 2 Department of Animal and Dairy Science, 425 River Road, University of Georgia, Athens, GA 30602, USA; johngonz@uga.edu

**Keywords:** poultry, enzyme, texture, myopathy, meat quality

## Abstract

**Simple Summary:**

With the advent of fast-growing big broiler chickens with high feed conversion ratios, the poultry industry is facing some meat quality issues. Woody breast myopathy is one such meat quality issue that renders the breast meat tough to touch when raw and is described as chewy and crunchy after cooking. One of the major reasons for the texture modification is the excess of collagen, especially with a high degree of cross-linking, in the breast muscle. Therefore, a research project was undertaken to examine a method to degrade the collagen and improve the tenderness of the meat. We mixed an enzyme, collagenase Type I, into the ground woody breast meat to determine its impact on collagen. We found that collagenase Type I can convert the insoluble collagen into soluble collagen. The study will help the poultry industry to develop marinades with collagenase to improve the texture of woody breast meat and increase its consumer acceptability.

**Abstract:**

Abnormal collagen infiltration in the *Pectoralis major*, breast muscle, of fast-growing big broilers has led to woody breast (WB) myopathy resulting in meat quality issues. Mechanisms to degrade the collagen were investigated to potentially improve WB texture. Freshly deboned WB fillets (*n* = 5 per trial; 3 trials) were ground and divided in to 25 g portions. Aqueous collagenase Type I solution (1 mL) from concentrations of 2.5, 5, and 10 mg/mL were incorporated in ground WB samples (*n* = 3 samples/treatment × 3 trials). Ground WB with 1 mL water acted as a control. All the samples were placed at 4 °C for 24 h and frozen at −80 °C. Control samples without any treatment or water addition (*n* = 3/trial) were frozen immediately upon grinding. Data collected on total (TC), soluble (SC), and insoluble collagen (IC) content was analyzed using one-way ANOVA with Tukey’s honestly significant difference (HSD) (*p* ≤ 0.05). Fresh WB fillets had TC, SC, and IC content of 19.5, 4.9, and 14.6 mg/g, respectively. The addition of collagenase decreased (*p* ≤ 0.05) the IC to 5.8 mg/g in the 10 mg/mL treatment after 24 h. Converting IC to SC using collagenase can potentially help the poultry industry to reduce WB toughness.

## 1. Introduction

Woody breast (WB), a myopathy observed in fast-growing big broilers, is affecting the US as well as the international poultry industry [1,2,3,4,5]. Tijare et al. reported a significant increase in collagen content in WB meat compared to the normal ones [5]. Similarly, Sihvo et al. concluded the hardness in WB meat can be due to increases in the amount of collagen [2]. Velleman and Clark showed WB meat from two strains of birds exhibited fibrosis with extensive extracellular collagen distribution, which was either cross-linked with parallel packing or diffused distribution [6]. Meat exhibiting the WB phenotype has a negative effect on visual appearance and overall consumer acceptance. In addition to consumer acceptance issues, meat with WB affects the functionality of myofibrillar proteins as indicated by lower water-holding capacity, marinade uptake (6.24% compared to 13.15% in normal fillets), higher cook loss (18.7% compared to 15.3% in normal fillets), and decrease in protein content (21.1% vs. 22.6%) and increased lipid content (2.94% vs. 2.36%), which negatively affects yield [7,8,9]. As a result, poultry processors have to sell the WB meat as low-quality meat for reduced price and incur significant losses [8,9]. 

Breaking down collagen using enzymes is a routine practice in the meat industry. Enzymes such as proteases (papain, bromelain) are extensively used by the beef industry to digest collagen and improve beef texture [10]. Historically, poultry meat contained low collagen content [8] and did not require enzymes to break down the collagen. With the rising incidences of WB meat having higher collagen content than the normal meat [8], the application of enzymes in poultry utilized for further processing can provide a solution to texture issues. Research was conducted to determine the effects of collagenase on the changes in collagen content (soluble, insoluble, and total collagen) of ground WB meat.

## 2. Materials and Methods 

### 2.1. Woody Breast Meat

Freshly deboned severe woody breast fillets (*n* = 5/trial × 3 trials) (approx. 500 g each) from 3 h post-slaughter fast-growing big broilers (3.6 to 4.1 kg live wt) were obtained from a local commercial poultry processor. The breast fillets were scored via manual palpation to select severe woody breast fillets. These fillets felt tough to the touch throughout and were stiff when held horizontal. Fillets were transported to the Auburn University Poultry Science Department under refrigeration (4 °C) and processed for further experimentation. Fillets were collected on three separate days where each day represented one trial.

### 2.2. Ground Meat Preparation

Severe WB fillets (*n* = 5) were ground together using a tabletop meat grinder (model Mini-32, Biro Manufacturing Corp., Marblehead, OH, USA). The meat was ground to reduce stearic hindrance and allow increased interaction between the substrate (collagen) and the enzyme. Ground meat (2500 g) was then aliquoted into six different treatments as follows: 1. untreated control (frozen immediately), 2. untreated control (frozen after 24 h refrigerated storage), 3. meat + 1 mL water, 4. meat + 2.5 mg collagenase/mL water, 5. meat + 5 mg collagenase/mL water, and 6. meat + 10 mg collagenase/mL water. Similar setup was repeated on the fillets collected on separate days, which represented independent trials.

### 2.3. Collagenase

Collagenase Type I was obtained from Worthington Biochemical Corporation (Lakewood, NJ, USA). The enzyme from three production batches was procured to be used in three separate trials. Collagenase solutions for treatments 4, 5, and 6 were prepared by solubilizing 12.5, 25, and 50 mg collagenase in 5 mL deionized water, respectively.

### 2.4. Treatment of Ground Meat

The meat aliquoted for each treatment was further subdivided into 25 g portions (n = 3 × 6 treatments) and placed in 50 mL plastic conical tubes. Each 25 g portion was treated as per the treatments 1 through 6. The 3 × 25 g portions of treatment 1 were frozen immediately in −80 °C freezer to prevent proteolysis and resulting effects on the collagen. Treatment 2 was kept under refrigeration (4 °C) for 24 h to allow any inherent proteolytic enzymes from degrading the collagen. In treatment 3, 1 mL water was added to compensate for the water being added in treatments 4 through 6. Enzyme solutions prepared as stated above were added (1 mL) to each of the 25 g meat portions. The enzyme solutions were mixed thoroughly in the meat using glass rods. Treatments 2–6 were placed in a refrigerator maintained at 4 °C for 24 h to allow for the enzymes to react with the collagen. 

### 2.5. Sample Analysis

Soluble and insoluble collagen were extracted and quantified using a protocol adapted from [11,12] as outlined by Ebarb et al. [13]. Briefly, 3 g of ground muscle tissue was incubated in Ringer’s solution at 77 °C for 80 min. After centrifugation at 2250× *g* for 12 min at 20 °C, the supernatant containing the soluble collagen fraction was separated from the insoluble collagen containing precipitate. Sulfuric acid was added (3 mL concentrated sulfuric acid to soluble portion; 30 mL 3.5 M sulfuric acid to the insoluble portion) and fractions were incubated at 105 °C for 16 h to hydrolyze the collagen. Extracts were diluted, filtered with Whatman 541 filter paper (Fisher Scientific; Waltham, MA, USA), and hydroxyproline determination was carried out using a BioTek Eon spectrophotometer [14] (Biotek Instruments Inc., Winooski, VT, USA) reading absorbance at 558 nm. Collagen content was determined by multiplying hydroxyproline content of the soluble fraction by 7.25 and the insoluble fraction by 7.52 [15]. Total collagen content was determined by adding the collagen fractions together.

### 2.6. Study Design and Statistical Analysis

The whole study was repeated in three independent trials. Each trial was conducted by collecting fillets from a different processing day to encompass the variation in collagen in the meat. The data was analyzed using one-way ANOVA with the treatments as fixed effects. Tukey’s HSD to was used to determine significant differences among the treatment means at *p* ≤ 0.05. 

## 3. Results

**Figure 1:**
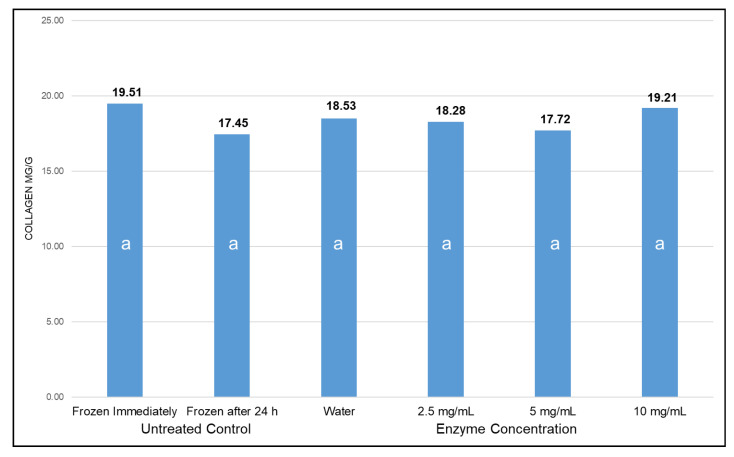
Effect of collagenase at different concentrations on total collagen (mg/g) in ground woody breast meat. Similar letters in the bar graph represent no significant differences among the treatments (*p* > 0.05).

**Figure 2:**
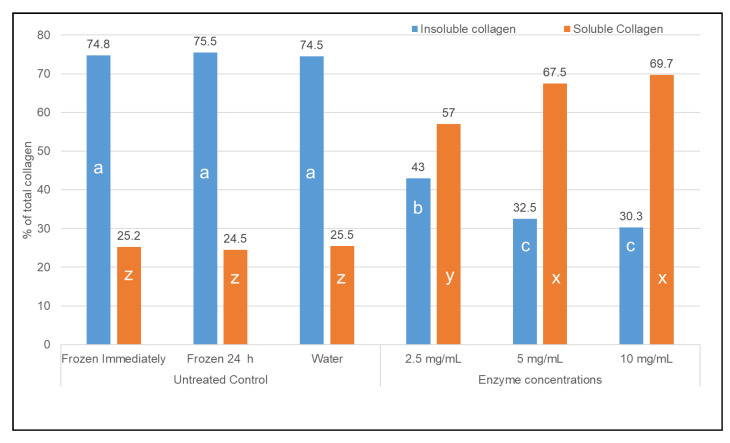
Effect of collagenase at different concentrations on insoluble and soluble collagen (% of total collagen) in ground woody breast meat. Letters a, b, and c represent significant differences in insoluble collagen while letter x, y, and z represent significant differences in soluble collagen among the treatments (*p* ≤ 0.05).

Total collagen of the freshly procured severe woody breast ground meat (Figure 1) was 19.51 mg/g of meat and did not change (*p* > 0.05) due to various treatments, including refrigerated storage at 4 °C with and without different enzyme concentrations. The insoluble collagen content of freshly ground severe woody breast meat (Figure 2) comprised of 74.8% of the total collagen. Refrigeration of the meat for 24 h and addition of water to compensate for the liquid added to the meat did not impact (*p* > 0.05) insoluble collagen content of the meat. The addition of collagenase at 2.5 mg/mL significantly reduced the insoluble collagen content of the meat compared to the untreated control samples to 43.0%. Insoluble collagen content decreased (*p* ≤ 0.05) to 32.5% with the increase in collagenase concentration to 5 mg/mL but did not decrease further (*p* > 0.05) with additional inclusion of the enzyme at 10 mg/mL. The 5 and 10 mg/mL treatments had less (*p* ≤ 0.05) insoluble collagen than the non-enzyme treatments. On the other hand, the total concentration of soluble collagen in freshly ground severe woody breast meat was 25.2% of the total collagen (Figure 2). Soluble collagen was not altered (*p* > 0.05) due to refrigeration for 24 h or inclusion of water. However, the soluble collagen content doubled (*p* > 0.05) with the addition of 2.5 mg/mL of collagenase. Further addition of 5 mg/mL of collagenase demonstrated an increase *(p* ≤ 0.05) in soluble collagen to 67.5%. The addition of 10 mg/mL of collagenase did not further increase (*p* > 0.05) the soluble collagen concentration compared to the 5 mg/mL treatment.

## 4. Discussion

Woody breast myopathy is associated with structural changes in the *pectoralis major* of the fast-growing big broilers. The breast muscle affected with WB demonstrates increased infiltration of collagen, especially Type I and III replacing myofibrillar proteins [16]. Further increased cross-linking in the collagen is observed with the age of the broilers leading to tough breast meat [16]. Maharjan et al. reported total collagen linearly increased with the age of the bird from 8.85 μg/mg in 21-day old broilers to 21.89 μg/mg in 56-day old broilers [17]. The data from Maharjan et al. [17] is similar to the current study where we detected a total collagen content of 19.51 mg/g of meat in 56-day old broilers. Compared to the current study, Welter et al. [18] reported much lower concentrations of collagen (3.89 mg/g of meat) in woody breast meat. These differences could be due to the selection of the WB severity. The breast fillets selected in the current study were extremely stiff, whereas Welter et al. [18] reported they selected moderate to severe breast fillets for their analysis. 

Total collagen was comprised of approximately 74% of insoluble collagen in the freshly ground, severe WB meat, indicating a higher degree of cross-linking between the collagen fibers. It should be noted that ground meat samples incubated at 4 °C for 24 h without the addition of enzymes did not show reduction in insoluble collagen content, indicating there was no significant inherent collagenolytic activity in the meat. The addition of Collagenase Type I at 2.5, 5, and 10 mg/mL resulted in collagenolysis in the meat samples as demonstrated with the decrease in insoluble collagen with a concomitant increase in soluble collagen (Figure 2). Collagenase Type 1 consists of collagenase, caseinase, clostripain, and trypsin-like enzymes (http://www.worthington-biochem.com/cls/cat.html: Accessed 7 August 2020). The current observations might be due to the natural intact collagen specific action of Type I collagenase, a product of *col*G gene, extracted from *Clostridium histolyticum* [19]. The crude collagenases might have enhanced activity in severe woody breast meat due to higher levels of Ca^2+^ [18], which are activators for these enzymes (http://www.worthington-biochem.com/cls/default.html: Accessed: 7 August 2020). 

Treatment with collagenase increased the solubility of the collagen while reducing the insoluble collagen content. Ebarb et al. [13] demonstrated that higher insoluble collagen content was directly proportional to the tough texture of *Longissimus lumborum* steak. The authors also stated that the increase in soluble collagen content due to aging increased the tenderness of the meat. Similarly, the enzymatic conversion of insoluble collagen to soluble collagen in WB meat will also impact its texture. 

## 5. Conclusions

In conclusion, the study successfully demonstrated the application of collagenase Type I at 2.5 to 5 mg/mL to convert insoluble collagen into soluble collagen of WB meat. Data also indicated inherent collagenase activity was absent in the severe WB meat analyzed in the study. Woody breast meat quality researchers should report soluble and insoluble collagen content instead of total collagen. It should be noted that the research was conducted in a ground meat system, and it provides a basis to conduct studies on whole muscle tenderization using collagenase Type I. Adding collagenase is a conceivable application that the poultry industry can consider using as a marination ingredient to reduce the implications of woody breast texture on value-added poultry products.
